# Medication Errors in the Southeast Asian Countries: A Systematic Review

**DOI:** 10.1371/journal.pone.0136545

**Published:** 2015-09-04

**Authors:** Shahrzad Salmasi, Tahir Mehmood Khan, Yet Hoi Hong, Long Chiau Ming, Tin Wui Wong

**Affiliations:** 1 School of Pharmacy, Monash University Malaysia, Sunway City, Selangor, Malaysia; 2 Department of Physiology, Faculty of Medicine, University of Malaya, Kuala Lumpur, Malaysia; 3 Faculty of Pharmacy, Brain Degeneration and Therapeutics Group, Pharmaceutical and Life Sciences CoRe, Universiti Teknologi MARA, Puncak Alam, Selangor, Malaysia; 4 Non-Destructive Biomedical and Pharmaceutical Research Centre, iPROMISE, Universiti Teknologi MARA, Puncak Alam, Selangor, Malaysia; Taipei Veterans General Hospital, TAIWAN

## Abstract

**Background:**

Medication error (ME) is a worldwide issue, but most studies on ME have been undertaken in developed countries and very little is known about ME in Southeast Asian countries. This study aimed systematically to identify and review research done on ME in Southeast Asian countries in order to identify common types of ME and estimate its prevalence in this region.

**Methods:**

The literature relating to MEs in Southeast Asian countries was systematically reviewed in December 2014 by using; Embase, Medline, Pubmed, ProQuest Central and the CINAHL. Inclusion criteria were studies (in any languages) that investigated the incidence and the contributing factors of ME in patients of all ages.

**Results:**

The 17 included studies reported data from six of the eleven Southeast Asian countries: five studies in Singapore, four in Malaysia, three in Thailand, three in Vietnam, one in the Philippines and one in Indonesia. There was no data on MEs in Brunei, Laos, Cambodia, Myanmar and Timor. Of the seventeen included studies, eleven measured administration errors, four focused on prescribing errors, three were done on preparation errors, three on dispensing errors and two on transcribing errors. There was only one study of reconciliation error. Three studies were interventional.

**Discussion:**

The most frequently reported types of administration error were incorrect time, omission error and incorrect dose. Staff shortages, and hence heavy workload for nurses, doctor/nurse distraction, and misinterpretation of the prescription/medication chart, were identified as contributing factors of ME. There is a serious lack of studies on this topic in this region which needs to be addressed if the issue of ME is to be fully understood and addressed.

## Introduction

Southeast Asia is a region of enormous cultural, economic and social diversity. It consists of eleven countries: Brunei, Cambodia, Indonesia, Laos, Malaysia, Myanmar, Philippines, Singapore, Thailand, Timor and Vietnam, collectively known as the Association of Southeast Asian Nations (ASEAN) [1]. The total population of this region is approximately 600 million (9% of the world’s population) with Indonesia being the region’s most populated country (comprising 40% of the total population of Southeast Asia) while Brunei is the least populated [2, 3].

The Southeast Asian countries possess diverse health systems which are at different stages of evolution. This is not surprising given the differing rates of demographic and epidemiological transitions, along with the cultural diversity of the nations residing in this region. Despite rapid developments, the health systems of Southeast Asia still lag behind those of Western countries.

According to the WHO, the average density of the health workforce in Southeast Asia is 4.3 per 1000 population, far less than that of Europe and the United States of American (US), which are 18.9 and 24.8 per 1000 population respectively [4]. This, unfortunately, holds true across all of the Southeast Asian countries: many, such as Vietnam, Myanmar, Laos and Cambodia fail to meet the WHO’s “basic” healthcare standard (2.28 skilled health workers per 1000 population), while Indonesia and Thailand barely reach this target; Malaysia and Singapore are exceptions to this, however [4]. Rapid but inequitable socioeconomic development, high population density, shortages in the healthcare workforce, coupled with enormous cultural diversity, have combined to pose great public health challenges for the national health systems of the Southeast Asian countries; one of these being the constant struggle to identify and minimize medication errors [5]. It is reported that unbalanced staff to patient ratios due to high population growth and shortages in healthcare professionals leads to long working hours without breaks, multitasking, an uncongenial environment and sleeplessness, all of which are important causes of skipping or violation of procedural steps [6].

Medication error (ME) is defined as *"any preventable event that may cause or lead to inappropriate medication use or patient harm while the medication is in the control of the health care professional*, *patient*, *or consumer*. *Such events may be related to professional practice*, *health care products*, *procedures*, *and systems*, *including prescribing; order communication; product labelling*, *packaging*, *and nomenclature; compounding; dispensing; distribution; administration; education; monitoring; and use"* [7]. It accounts for one third of preventable drug-related harm and is the eighth leading cause of death in the US with more than 98,000 mortality annually, exceeded those from car accidents, breast cancer, or AIDS [8].

Although ME is a worldwide issue [9], the majority of the studies on this topic have been carried out in developed nations such as North American and European countries, while the issue has been relatively neglected in Southeast Asia. In this systematic review of the literature, we focused on the types and prevalence of ME in Southeast Asian countries. This broad focus represents an initial attempt to understand the scope of ME in this region, recognising that the types and prevalence of ME reported in US and European based studies may not closely reflect the situation in Southeast Asia due to fundamental differences in key aspects of the healthcare system in this region, such as the ratio of healthcare professionals to the general population and drug prescribing patterns. These differences are expected to have a direct influence on the general trend, prevalence and types of MEs in Southeast Asia since, as the Institute of Medicine argued in its report entitled “Crossing the Quality Chasm: A New Health System for the 21st Century”, in most cases the individual’s performance is governed by the system and MEs need to be seen as symptoms of system failure rather than as the result of an individual’s failure. In complex healthcare systems, the number and types of processes involved in handling medications itself leads to error as a failure in any one step of the process permits medication errors to occur [10, 11]. This review, therefore, aimed systematically to identify and review studies on the incidence and types of MEs in Southeast Asian countries in order to identify common MEs and estimate its prevalence in this region.

## Methods

### Search strategy

**Fig 1:**
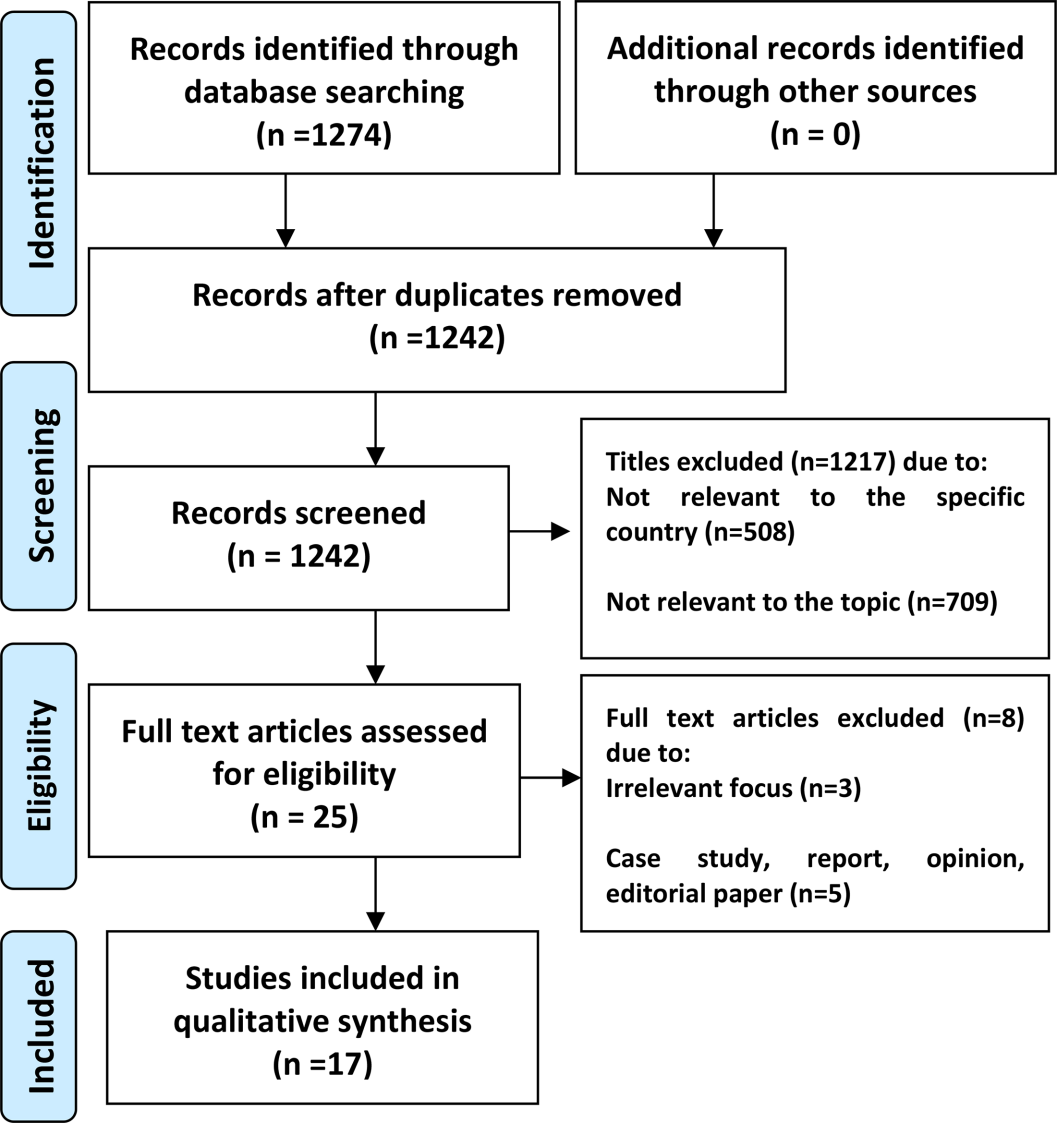
PRISMA diagram demonstrating the search strategy and its results.

The studies undertaken on MEs were systematically reviewed in December 2014. The exact electronic search strategy is outlined in Fig 1, below. The search strategy included human studies of all languages, and all types of trials on patients of all ages (Refer to S1 PRISMA Checklist)

### Search terms

The following words were combined with “Southeast Asia” and appropriate country names, using the Boolean operators (“AND” & “OR”): medication error(s), prescribing error(s), dispensing error(s), administration error(s), documentation error(s), transcribing error(s), medication mistake(s), drug mistake(s), prescribing mistake(s), dispensing mistake(s), administration mistake(s), transcribing mistake (s), wrong medication, wrong drug (s), wrong dose(s), wrong route of administration, wrong medication history taking, wrong calculation(s), physician(s), pharmacist(s) and nurse(s). The detailed description of the search strings used and the results obtained can be found in Table 1.

**Table 1:** Search terms used.

Search terms	Search engine	Results	Chosen
**((Medication error) AND Southeast Asia) AND prescription error**	Pubmed	03	02
**((((Malaysia) OR Thailand) OR Brunei) OR Singapore) AND medication error**	Pubmed	07	05
**((((((((Medication error) AND Timor)) OR ((medication error) AND Indonesia)) OR ((medication error) AND Myanmar)) OR ((medication error) AND Cambodia)) OR ((medication error) AND Philippines)) OR ((medication error) AND Laos)) OR ((medication error) AND Vietnam)**	Pubmed	09	01
**'error'/exp OR error AND ('medication'/exp OR medication) AND ('safety'/exp OR safety) AND ('Southeast Asia'/exp OR 'Southeast Asia') AND [humans]/lim AND [english]/lim AND [2010–2015]/p**	Embase	37	11
**'medication'/exp OR medication AND ('error'/exp OR error) AND ('prevention'/exp OR prevention) AND ('Southeast Asia'/exp OR 'Southeast Asia') AND [humans]/lim**	Embase	30	04
**(((prescribing error) OR medication error) AND ASEAN) OR association of Southeast Asian nations**	Pubmed	48	0
**((AB medication error OR AB dispensing error OR AB prescribing error OR AB transcribing error) AND (Southeast Asia OR ASEAN OR Philippines OR Indonesia OR Myanmar OR Laos OR Timor OR Cambodia OR Malaysia OR Singapore OR Vietnam OR Brunei))**	EBSCOhost (Medline)	14	02
**((Medication error) OR (prescribing error OR dispensing error) OR (administration error OR reconciliation error) OR (transcribing error OR patient safety)) AND loc(Southeast Asia)**	Proquest Central	192	0
**medication error OR prescribing error OR dispensing error AND Southeast Asia**	EBSCOhost (CINAHL Plus)	866	0

### Inclusion/exclusion criteria

The following types of studies were included; randomized controlled trials, non-randomized controlled trials, longitudinal studies, cohort or case–control studies, and descriptive studies. There was no limitation imposed on the year of publication of the studies. Reviews, letters, case studies, conference papers, opinions, reports or editorial papers were not included, however.

### Quality assessment

All identified studies were reviewed in terms of quality and were assessed according to thirteen criteria adapted from Alsulami et al. [9]. These were as listed below:

1. Aims/objectives of the study clearly stated.
2. Definition of what constitutes a medication error.
3. Error categories specified.
4. Error categories defined.
5. Presence of a clearly defined denominator.
6. Data collection method described clearly.
7. Setting in which study conducted described.
8. Sampling and calculation of sample size described.
9. Reliability measures.
10. Measures in place to ensure that results are valid.
11. Limitations of study listed.
12. Mention of any assumptions made.
13. Ethical approval.

Studies that fulfilled less than seven of these criteria were considered poor quality, 7–10 criteria met were considered average quality and studies that met more than 10 criteria were of good quality.

## Results

### Search results

**Fig 2:**
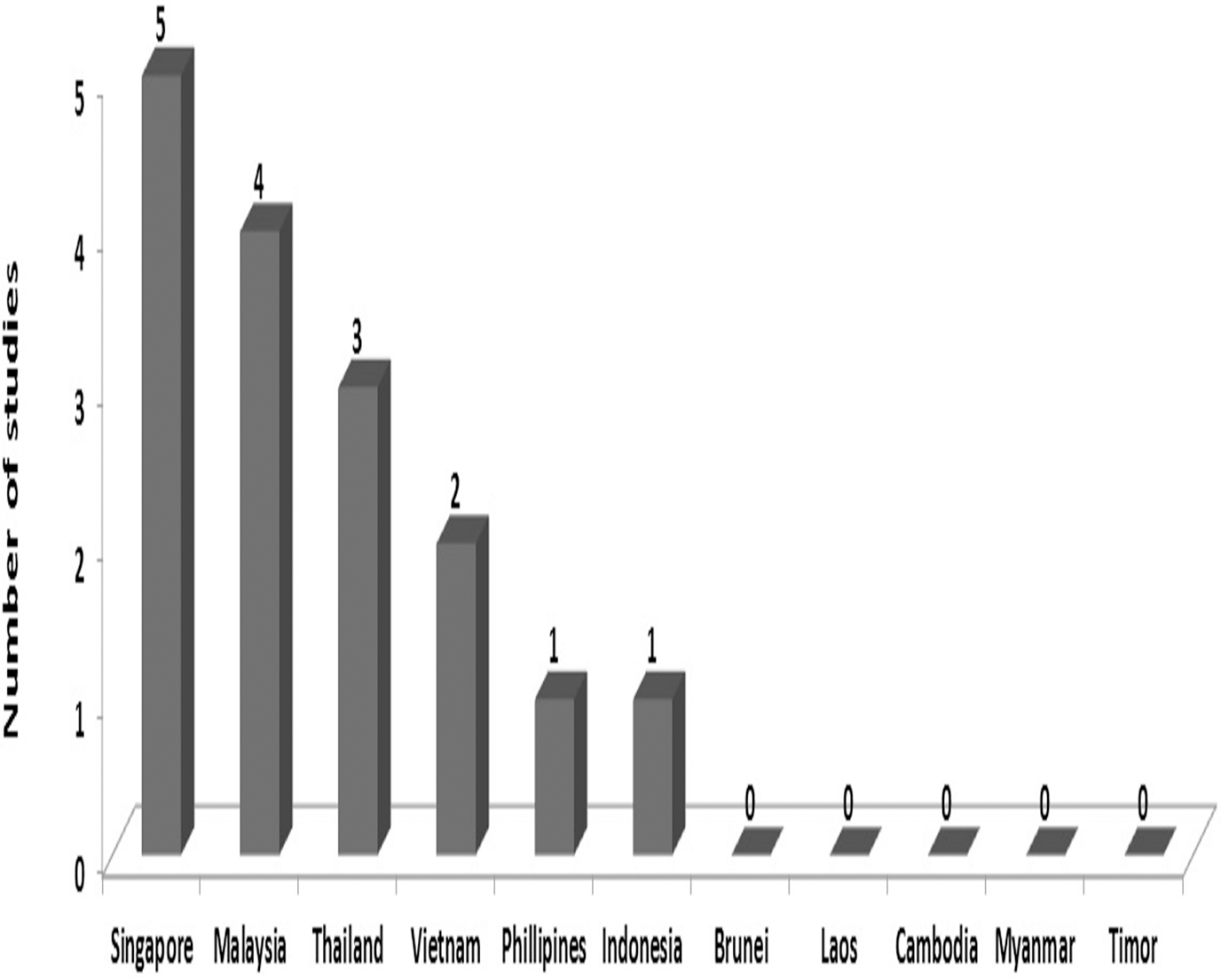
Number of studies done in each country on ME in Southeast Asia.

Fig 1 demonstrates the search strategy used and its results; 17 articles were retrieved and included in this systematic review. Fig 2 demonstrated the number of studies obtained from each of the Southeast Asian countries.

### Quality assessment of studies

**Fig 3:**
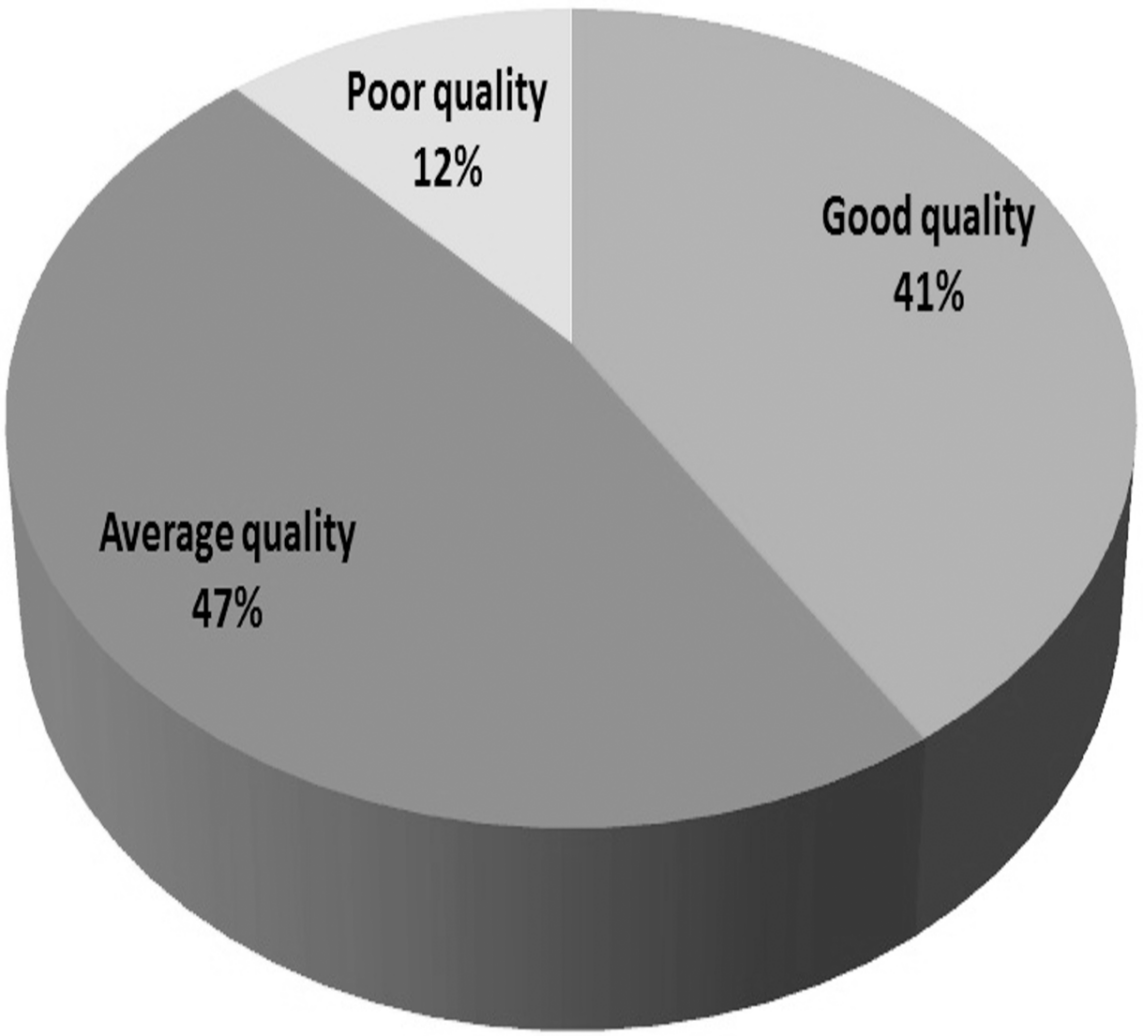
Quality classification of the included studies.

Using the criteria adopted from Alsulami et al. [9], the quality of the included studies was assessed, then, based on the quality assessment results, they were each classified into the three quality assessment categories (poor, average and good quality), as presented in Fig 3.

### Types of medication error studied

**Fig 4:**
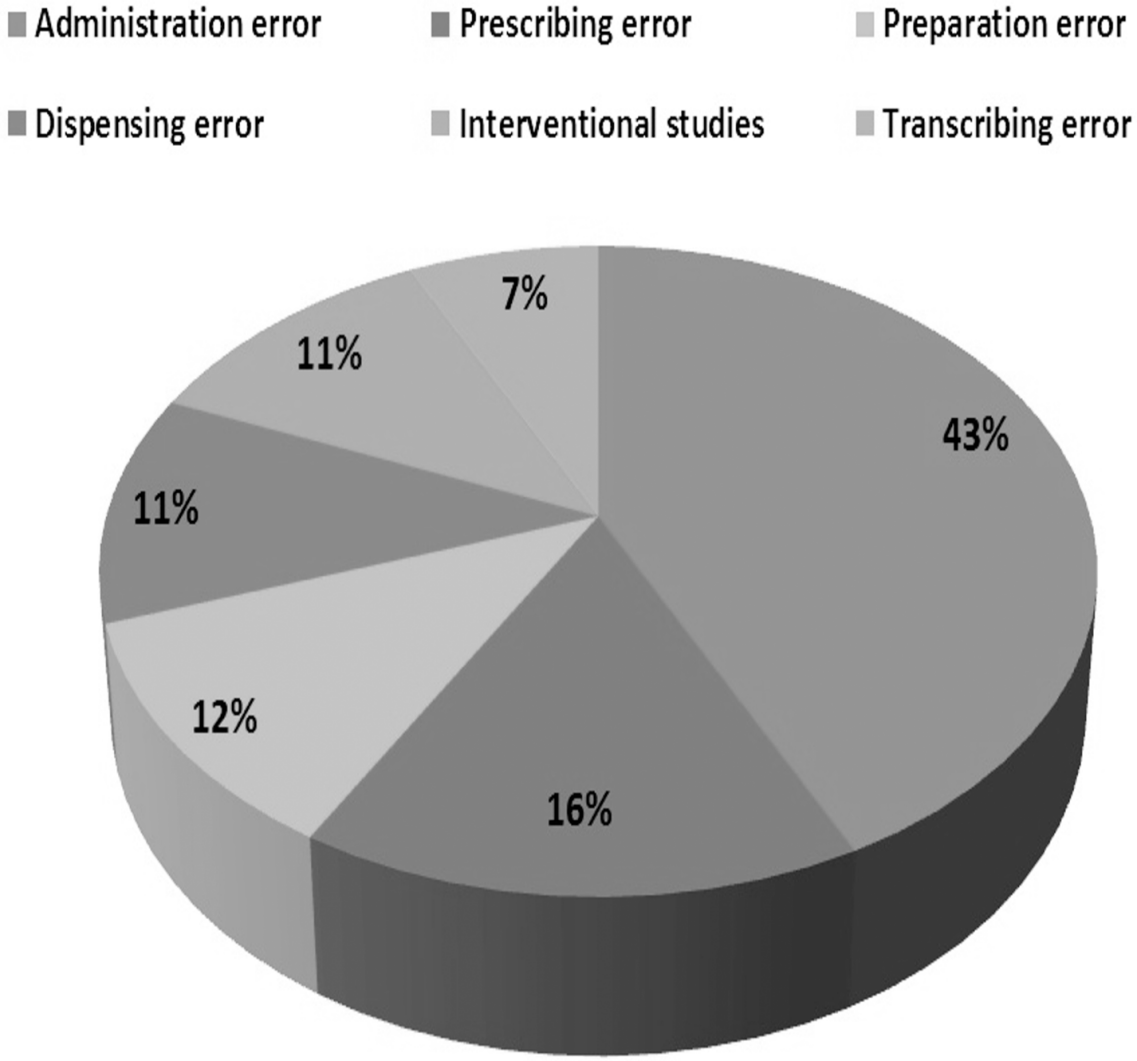
Study classification according to error types.

Study classification according to error types is presented in Fig 4.

### ME classification

The included studies used different methodologies, making comparison between the error incidence outcomes difficult. The results were, therefore, classified based on the stage of the medication treatment process in which the error occurred, such as: dispensing, prescribing, transcribing, administration, etc.

### Characteristics of the included studies

The characteristics of the included studies are summarised in Table 2.

**Table 2:** Characteristics of the included studies.

**Studies done on administration error**								
**#**	**Setting**	**Methodology**	**Study duration**	**Population**	**Sample**	**Results**	**Total number of erroneous administrations**	**Reference**
1	Malaysia (Paediatric ward of a teaching hospital)	Direct observational study	10 days over 10 weeks	50 patients age less than 16 years old; admitted to the pediatric ward	857 administrations	Incorrect dose form: 2% Incorrect time: 30% Incorrect technique: 9% Unauthorized drug: 7% Omission error: 17% due to out of stock Incorrect dose: 12% Incorrect preparation: 27%	11.7% (100 of the 857 administrations observed)	[12]
2	Malaysia (tertiary care hospital)	Prospective observational study	3 months	Patients hospitalized in all 24 wards of the hospital	349 IV drugs which were prepared and administered by the staff nurses to the patients.	Wrong time: 42.1% Wrong technique: 19.5% Wrong administration rate: 85.1%	88.6% (302 of the 349 administrations observed)	[13]
3	Malaysia (haematology ward of a teaching hospital in Malaysia)	Prospective study that involved direct, undisguised observations of drug administrations	15 days	Patients hospitalized in the heamatology ward of the hospital	1118 total opportunities for error	Incorrect drug: 0.7% Extra dose: 2.2% Administration of expired medications: 2.2% Incorrect rate: 5.9% Omission: 10.4% Incorrect dose: 10.4% Incorrect drug preparation: 10.4% Unauthorized drug: 14.1% Incorrect technique: 16.3% Incorrect time: 25.2%	11.4% (127 of the 1118 administrations observed)	[14]
4	Singapore (public sector and private practice anaesthesiologists in Singapore)	Survey	1 month	174 anaesthetists, trainees and specialists in public institutions or in private practice	350 survey forms	Misidentification of the ampoule: 53% Misidentification of syringes: 45%	45.4% (159 of the 350 errors/near misses reported)	[15]
5	Singapore (two acute care hospitals)	Descriptive, prospective design	12 weeks	140 registered full time nurses	21043 opportunity for error (doses given or doses orders but omitted) An opportunity for error included any dose given plus doses ordered to be given but omitted	Of the 140 participants, only 10% (14/140) were not observed to encounter any distractions during medication administration, while 90% (126/140) were distracted during the observations	45.4% (5024 out of the 21043 opportunities for error)	[16]
6	Vietnam (2 public hospitals)	Direct observational study	7 days	Diabetic patients in ward	229 insulin doses (204 subcutaneous and 25 infusions)	Delayed dose: 10.4% Early dose: 7.4% Administration technique error: 3.1% Omission: 2.6%	28.8% (66 of the 229 insulin doses)	[17]
7	Vietnam (6 wards in 2 urban public hospitals)	Prospective observational	3 months		5271 doses administered	Wrong administration technique: 23.5% Wrong preparation technique: 15.7% Omission: 2.3% Wrong dose: 1.8%	39.1%	[18]
8	Indonesia-Bali (Geriatric ward in a public teaching hospital)	Prospective study	20 weeks	Geriatrics (>60 years old) patients in ward	7662 doses	Administration errors: 59%	20.4% (1,563 medication errors of 7,662 drug doses reviewed)	[19]
9	Philippines (University-based tertiary hospital)	Questionnaire		Junior and senior nursing students who routinely administer medications within a university-based tertiary hospital	329 questionnaires	Missed dose: 41.94% Wrong time: 40.32%	18.8% (63 out of the 329 respondents)	[20]
10	Thailand (7 university hospitals, 5 tertiary care hospitals, 4 secondary care hospitals, 4 primary care hospitals)	Prospective data collection	18 months	Patients anaesthetized in 20 participating hospitals in Thailand	202699 anaesthesia cases	Wrong drug: 48.8% Incorrect dose: 29.3%	0.02% (41 of the 202699 cases)	[21]
11	Thailand (Queen Sirikit National Institute of Child Health)	Retrospective study (screening medication errors documents and reports)	15 months	Medical records from September 2001 to November 2002	Medication errors in ward documented in standard reporting forms based on the 32106 admissions	Administration error: 15.22% Wrong time: 2.17% Omission 1.24% Wrong strength: 1.86% Unauthorized drug: 0% Wrong patient: 2.48% Extra dose: 3.73% Wrong dose form: 3.73%	1% (322 of the 32105 admissions medical report)	[22]
**Studies done on dispensing error**								
1	Indonesia-Bali (Geriatric ward in a public teaching hospital)	Prospective study	20 weeks	Geriatrics (>60 years old) in ward	7662 doses	Dispensing errors: 14% Omission: 39.6%	20.4% (1,563 medication errors of 7,662 drug doses reviewed)	[19]
2	Thailand (Queen Sirikit National Institute of child health)	Retrospective study (screening ME documents and reports)	15 months	Medical records from September 2001 to November 2002	32105	Dispensing: 34.78%	1% (322 errors of the 32105 admissions)	[22]
**Studies done on prescribing error**								
1	Malaysia (outpatient pharmacy in a teaching hospital in Kelantan)	Retrospective study. (screening prescriptions)	1 month	Geriatrics at the outpatient pharmacy	1601 prescriptions for geriatrics	Pharmaceutical (stability, ingredient, technique): 0.99% Clinical errors (dose,frequency, interaction,allergy): 8.68%	25.15% (403 of the 1602 prescriptions)	[23]
2	Indonesia (Geriatric ward in a public teaching hospital in Bali)	Prospective study	20 weeks	Inpatient geriatrics (>60 years old)	7662 doses	Prescribing errors: 7%	20.4% (1,563 of the 7,662 drug doses reviewed)	[19]
3	Singapore (Paediatric unit in a university teaching hospital)	Prospective cohort study	4 months	Children (<16 years of age) at the outpatient clinic, emergency department and at discharge from the inpatient service	4274 paediatric prescriptions	Under-dose: 64% No frequency specified: 21%Overdose: 8%	19.5% (833 of the 4274 prescription screened)	[24]
4	Thailand (Queen Sirikit National Institute of child health)	Retrospective study (screening ME docs and reports)	15 months	Medical records from September 2001 to November 2002	32105	Prescription error: 35.4% Wrong dose: 25.78% Wrong choice: 3.73% Known allergy: 0.62%	1% (322/32,105 admissions)	[22]
**Studies done on transcribing error**								
1	Malaysia (outpatient pharmacy in a teaching hospital (HUSM) in Kelantan)	Retrospective study. (screening prescriptions)	1 month	Geriatrics at the outpatient pharmacy	1601 prescriptions for geriatrics	Miswriting patient particulars: 70.22%	25.15% (403/1602 prescriptions)	[23]
2	Indonesia (Geriatric ward in a public teaching hospital in Bali)	Prospective study	20 weeks		7662 doses	Transcription errors: 15%	20.4% (1,563/ 7,662 drug doses reviewed)	[19]
**Studies done on preparation error**								
1	Malaysia (tertiary care hospital)	Prospective observational study	3 months	Patients hospitalized in all 24 wards of the hospital	349 IV drugs prepared and administered by nurses	Preparation errors: 32.8% Wrong amount of diluents: 54.5%	88.6% (302 of the 349 administrations observed)	[13]
2	Vietnam (Two large public hospitals in Vietnam)	Direct observational study	7 days	Diabetic patients admitted in wards	229 insulin doses (204 subcutaneous and 25 infusions)	Incorrect preparation technique: 22.7%	28.8% (66 of the 229 insulin doses)	[17]
3	Vietnam (two urban public hospitals in Vietnam)	Prospective observational	3 months	6 wards	5271 oral and IV doses administered	Wrong preparation technique: 15.7%	39.1% (2060 of the 5271 administration)	[18]
**Studies done on reconciliation error**								
1	Singapore (Tan Tock Seng Hospital)	Descriptive	NA	5100 patients admitted	Reconciliation forms created by pharmacy staff for each patient admitted	Transcription error: 36.5% Prescribers missing out medications from their list: 61.65% Wrong or incomplete regimen: 25.4%	18.13% (925 of the 5100 patients admitted)	[25]

### Administration error

Medication administration error is defined as any discrepancy between the medicine given to the patient and the prescriber's medication order as written on the patient's chart or manufacturers' preparation/administration instructions [26]. Eleven of the seventeen studies discussed administration errors. The reported administration error rates ranged from 15.2%, to 88.6% [13, 19, 22]. “Wrong time” (early or delayed doses) was the most frequently cited type of administration error [12–14, 17, 20, 22], along with omission error, where the dose was not administered at all [12, 17, 20, 22, 23]. The next most frequently mentioned type of administration error was “wrong dose” [12, 14, 21, 22].

Ong et al. [13] who focused on IV drug administration errors reported that the most frequent administration errors were mistakes in the technique of IV administration, along with the medication being administered at the wrong rate (usually too fast).

Chua et al. [12] who investigated administration error in paediatric wards, reported that administration errors are more prevalent in oncology wards since the medicines are more complex. The same study also mentioned that liquid dose forms are more prone to administration error as compared to solid dose forms because of the measurement errors that occur during the measurement of the volume required for liquid doses.

The other frequently reported reasons behind administration error were: heavy workload of nurses (due to nurse shortage) [12–14, 19, 20, 27], lack of knowledge [12, 14], stock shortage [12, 23] and calculation error [12, 14].

### Dispensing error

Dispensing errors happen when the medication dispensed/delivered by the pharmacy is not compatible with the order written in the prescription by the doctor [28]. Of the seventeen studies, four investigated dispensing error; with one retrospective, one prospective and two observational studies. Ernawati et al. reported an error incidence of 14%, Nguyen HT et al. reported a rate of 22.7%, while Ong et al. and Sangtawesin et al. reported roughly equal error incidence (32.8% and 34.78% respectively). Ernawati et al. reported omission as the main type of dispensing error followed by, in order of decreasing frequency, labelling errors, wrong quantity of drug, wrong dose, duplication, wrong drug, drug dispensed although not ordered, wrong dosage form and wrong patient. Additional types of dispensing error mentioned by Ong et al. were: wrong diluents or wrong amount of diluents, exceeding stability time after reconstitution, improper mixing.

### Prescribing error

Prescribing error is defined as any error in the process of prescribing the medication that leads to (or has the potential to lead to) patient harm [29]. The error rates reported varied greatly; the highest rate of prescribing error was reported by Sangtawesin et al., which was 35.4%, while the lowest error rate was 7% reported by Ernawati et al. The wrong dose was the most frequently mentioned type of prescribing error [19, 22–24]. Goh et al., however, stated that under-dosing was significantly more prevalent that overdosing and also that paediatricians made fewer prescribing mistakes than non-paediatricians (18.4% vs 25.1%) [24].

### Preparation error

Errors that occur during the preparation of a medicine could either happen in the pharmacy—for example when the pharmacist prepares an incorrect dilution for an oral syrup, or by the nurse in the ward when reconstituting an intravenous solution or crushing modified release tablets for oral tube administration. Some of the studies, however, did not really specify whether these errors occurred in the pharmacy or in the ward; in this review, therefore, any medication error that occurred in the process of preparation were classified as “preparation error” regardless of the health-professional responsible for it. Three of the seventeen included studies focused on preparation errors by either the pharmacist or the nurse. Two of these were conducted in Vietnam [17, 18] and one in Malaysia [13]. The common types of errors that happened in the preparation stage were use of the wrong technique or the wrong diluents.

### Transcribing error

According to Fahimi et al. *“Transcription error is a specific type of medication error and is due to data entry error that is commonly made by the human operators”* [30]. Two of the included studies investigated transcribing error [19, 23]; one in Malaysia and the other in Indonesia, with very different error rates reported: 15% [19] versus 70.22% [23]. Ernawati et al. reported that 35.2% of these errors involved drugs needed by patients not being transcribed either onto the medication chart or drug order form, or into the nurse’s log book; resulting in seven drug omissions in the administration stage and two delayed administrations.

### Reconciliation error

The Australian Commission on Safety and Quality in Healthcare defines medication reconciliation as *“a formal process of obtaining and verifying a complete and accurate list of each patient’s current medicines”*. Unfortunately there was only one study focused on errors that occur in this stage of patient care. This was performed in Singapore and reported a transcription error rate of 36.5%, which was mainly due to prescribers missing out medications from their list (61.6%) and an incorrect or incomplete regimen being transcribed (25.4%) [25].

### Interventional studies

Three studies investigated interventions used to reduce MEs (Table 3). Sanguansak et al. examined the use of formulary script instead of handwritten prescriptions and reported a significant decrease in prescribing errors such as drug name error, incorrect strength and incorrect route [31]. Nguyen et al. investigated the effect of a clinical pharmacist led training programme on intravenous medication errors, with a significant reduction of ME (from 64.0% to 48.9%) being observed in the intensive care unit (ICU) in cases where education had been provided by the pharmacist. Not all the interventional studies showed positive outcomes, however. In the study performed by Choo et al., an inpatient electronic medication record system was the intervention used to reduce ME, but the system was found to have little effect [32].

**Table 3:** Characteristics of the interventional studies included.

Setting	Methodology	Study duration	Sample	Intervention	Results	Reference
Thailand (Ophthalmology clinic in Srinagarind Teaching Hospital)	Non-randomized interventional	5 months	4349 handwritten prescriptions	Formulary script instead of handwritten prescriptions	**Handwritten vs formulary script** Legibility (Handwritten:16.1%; formulary script: 0.1%)(p<0.001) Incomplete (Handwritten:16.1%; formulary script: 0.1%) (p<0.001) Abbreviation (Handwritten: 3.1%; formulary script: 0.3%)(p<0.001) Ambiguous errors increased with formulary scripts (Handwritten: 0.6%; formulary script: 2.5%) (p<0.001) Accuracy errors (Handwritten: 0.8%; formulary script: 0.6%) (p = 0.21)	[31]
Singapore (Acute care hospitals)	Interventional retrospective	1 year	14000 hospitalized patients	Electronic ward medication record system	**Paper based system ME:** pre: 0.47 of the 1000 patient post: 0.41 of the 1000 patient **Electronic medication record ME:** Pre: 1.19 of the 1000 patient post:1.19 of the 1000 patient The mean incidence difference of 0.72 in medication errors was statistically significant between the two hospitals (95%, CI: [0.56, 0.88]).	[32]
Vietnam ICU and a post-surgical unit at major public hospital	A controlled, prospective, interventional study	2 weeks	1204 IV doses	A clinical pharmacist led training programme	**Post-surgical unit (No educational intervention)** No change in the prevalence of clinically relevant errors (57.9% vs 64.1%) p = 0.132 **ICU (With educational intervention)** ME decreased significantly from 64.0% to 48.9% (p < 0.001)	[27]

### Types of errors

Medication errors are typically classified into a few broad groups according to the stage at which the ME occurred (such as dispensing error, administration error, etc.). Although this approach can be useful, it does not help much when it comes to the prevention of MEs since it is important to know the exact types of errors that occur in practice. In the Southeast Asian countries, the wrong dose was the most common type of error [12, 14, 17, 19, 21–24, 31], with the reported rate of dose error—ranging between 12% [12] and 72% [24]. Other types of errors reported were:

- Omission error [14, 17, 19, 20, 22, 25]
- Incorrect time [12–14, 20, 22]
- Wrong drug [14, 15, 17, 21, 22]
- Incorrect administration technique [12–14, 17]
- Wrong dose form [12, 22, 25]

### Medications involved

Most studies did not mention the medications involved in the ME, although some did mention the name of the drug, while some others mentioned the drug class. The most frequently reported class of drugs related to ME was antibiotics [12, 13, 21, 22]. Other common medicines involved were opioids [15, 21], corticosteroids [12, 22] and muscle relaxants [15, 21].

### Severity of the medication errors

Unfortunately, in most of the studies, the clinical consequences of the reported MEs were not investigated. Nguyen et al. reported that 23.5% of the doses were judged to have potentially moderate outcomes and 3.5% potentially severe outcomes. Sangtawesin et al., who classified MEs in term of their severity, reported two clinically significant MEs, an over-dosage and a wrong dose for administration [22]. Thanoo et al., meanwhile, reported that 34.1% (14 out of 41 incidents) of MEs led to short term, mild to severe physiological effects, with all of the affected patients making a complete recovery, apart for one who died [21].

Generally speaking, however, the identified MEs led to interventions by researchers due to ethical reasons and hence did not lead to any clinically significant consequences for the patient.

### Factors contributing to medication errors

The factors contributing to MEs were reported in 15 studies [12–16, 19–25, 27, 31, 32]. The most common ones are listed below; it is important to remember, however, that MEs usually arise from poorly designed work environments and systems rather than the individual performance of a single practitioner [33].

- Staff shortage/high workload [12–14, 19–21, 27]
- Nurse/doctor distraction [12, 31, 32]
- Incorrect interpretation of prescription/medication chart [12, 14, 31]
- Lack of knowledge [12, 14]
- Lack of experience [20, 24]

It must be noted, however, that, while not the focus of this review, patients themselves may also contribute to the incidence of medication errors due to a number of reasons such as forgetfulness, lack of cooperation or confusion.

### Recommendations

The following are the recommendations collected from all the included studies which would be of use to decision-makers involved in reducing MEs in Southeast Asian countries:

- Educating patients/ staff [13, 14, 17, 20, 22, 23]
- Double-checking by nurses and pharmacists [13, 14, 17, 21–23]
- Having a clinical pharmacist in the ward [14, 19, 23]
- Appropriate labelling by manufacturers and pharmacists [15, 21]
- Administration time should be planned such that not all patients in a ward take their medications at the same time [12, 14]
- Ensuring an adequate, timely supply of medications to wards [12, 17]
- Improving patient/staff ratio [22, 27]
- Taking measures to reduce nurse’s distraction [16]

## Discussion

The number of studies done on ME in this region was unfortunately very limited (17 only), and of the eleven countries that make up Southeast Asia only six had reported data on ME. Most of the studies focused on administration and prescribing error. Furthermore, our findings highlight that the studies undertaken on ME in Southeast Asia are not only limited in quantity but also quality; over half of the included studies (59%) fell in the “average” or “poor” quality class with only 41% being “good quality” studies. Although one may speculate that the type, complexity, dose or dose form of the medicines may each be contributing factors to the incidence of ME, most studies did not mention the medications that were involved in MEs. Furthermore, the clinical consequences of the ME were not investigated by the majority of the studies since most of the errors were, for ethical reasons, caught by researchers and prevented from affecting the patient, making it difficult to assess the overall clinical impact of such errors. A better picture will become apparent if more studies are conducted on this topic. Certainly, it is evident from this systematic review that, just as in developed countries, ME is an area of concern in Southeast Asia.

Medications are the cornerstone of care provision. Although the safe use of medications can improve and save the lives of millions, errors in the use of these substances can lead to equally significant consequences. To our knowledge, no previous systematic review has evaluated MEs in Southeast Asia. With this systematic review, therefore, we attempted to identify and review the studies done on ME in Southeast Asia in order to gain an insight into the extent of the issue in this region. This is important since, apart from harming people physically and psychologically, and in some cases taking their lives, MEs result in a massive cost burden. For example, their approximate cost in the US is USD$17-$29 billion per annum [6]. MEs also lead to consequences beyond what money can repair; they can seriously damage public confidence and trust in medical services, and they affect the whole of society through lower productivity and decreased levels of population health [8]. Health authorities around the world have, therefore, always aimed to identify and minimize MEs [34]. In parts of Southeast Asia, however, the resources and the relevant governmental effort are still lacking. Table 4 summarizes the existing systems for reporting a medication error in Southeast Asian countries. It must be noted that the practice of a systematic method to ensure patient safety is relatively new for Cambodia. Most health professionals in this country are not very familiar with the concept of pharmacovigilance [35]. On the other hand, the Ministry of Health of Thailand has a good written policy of patient safety with well-planned strategic goals to minimize ME [36].

**Table 4:** ME reporting system available in Southeast Asia countries

Country	The ME reporting system
**Cambodia**	-The national pharmacovigilance system was established in 2008 following establishment of the Cambodian pharmacovigilance center in 2008 [35]
	-A participants of the WHO international drug monitoring program [35]
**Thailand**	-Pharmacovigilance system in Thailand was establishment 1983 under the Food and Drug Administration [35].
	-Member of international medication safety network [37]
	-Participant of the WHO international drug monitoring program [35]
**Malaysia**	-The Pharmaceutical Services Division has embarked on a reporting system called the Medication Error Reporting System under Malaysian Patient safety council (MPSC) [36]
	-Participant of the WHO international drug monitoring program [35]
**Singapore**	-Participating in the WHO international drug monitoring program [35]
	-Member of international medication safety network [37]
**Vietnam, Brunei, Indonesia, Philippines**	Participating in the WHO international drug monitoring program [35]

### Administration error

The reported administration ME rates ranged from 15.22% to 88.6%. The most frequent types of administration error reported were; wrong time, omission error and wrong dose. Staff shortage and hence heavy workload for nurses, as well as doctor/nurse distraction were identified as contributing factors for this type of ME. Choo et al., who considered nurse distraction to be the main reason behind administration error, reported the following as the main sources of nurse distraction; (i) physicians; (ii) other personnel; (iii) other patients; (iv) visitors; (v) telephone calls; (vi) missing medications; (vii) emergency situations; (viii) conversations; and (ix) external noise [16]. Reducing the nurse to patient ratio would probably be the most effective strategy to reduce administration error as it not only reduces the workload but also reduces distraction by ensuring that there is sufficient staff to allow each nurse to concentrate on one task at a time. The more complex medications such as oncology medication and parenteral medications were reported to have a higher rate of administration errors, suggesting that nurses administering such medications may need extra training and education.

### Prescribing error

Of the four studies focused on errors occurring at the prescribing stage, two had geriatrics as their population [19, 23] while the other two focused on paediatrics [22, 24]. Wrong dose was the most frequently mentioned type of prescribing error which involves both under-dosing and overdosing and was reported in all four studies [19, 22–24]. Sanguansak et al., who studied the impact of pre-printed prescriptions in an ophthalmic clinic, reported illegibility and incomplete information as the primary issue with prescriptions, and demonstrated that the use of pre-printed prescriptions significantly reduced errors in drug name, drug route and drug strength [31].

Overall, a number of solutions were suggested by the included studies, the most common one being staff/patient education because most of the underlying causes of ME can be attributed to a lack of awareness. Having a clinical pharmacist in the ward is one of the most proven approaches by which hospital staff and patients can be educated. Clinical pharmacists possesses an extensive knowledge of medication, are especially trained in therapeutics and are in the best position to detect and correct MEs; Kucukarslan et al. showed that having a clinical pharmacist on the medicine team reduced preventable adverse drug events (ADEs) by 78% [38]. Rothschild et al. conducted 226 observation sessions in which pharmacists reviewed 17,320 medications ordered/administered to 6,471 patients. In this study, 504 recovered medication errors were reported, with 47.8% of these errors being serious and 4.6% being potentially life-threatening [39].

### Medical error reporting systems

Clinical pharmacists are not only able to educate staff and detect and correct errors, they can also help to reduce MEs on a much larger scale by using Medical error reporting system to report errors so that they can be used by authorities for appropriate policy and decision making. The lack of data on ME from almost 50% of the countries in Southeast Asia is clear proof of the weaknesses in the medication error reporting system in the region. ME reporting is the most essential part of any strategy to reduce MEs, since learning from previous healthcare system failures assists in identifying root causes, which is in turn vital in reducing MEs [33, 40]. ME reporting provides valuable information allowing the situation to be better analysed, behaviours to be correctly predicted, and systems to be designed to be more safe and reliable and thus to prevent MEs. ME reporting highlights the areas of vulnerability in care provision and helps prevent repeat errors. MEs, however, are seriously under-reported around the world, including in Southeast Asia, since healthcare professionals (HCPs) are reluctant to report errors for reasons such as: fear of legal consequences, public embarrassment, disciplinary action, loss of credibility and lack of awareness about the importance of reporting. Things are changing, however: the implementation of non-punitive systems along with education has helped HCPs understand that not reporting errors imposes a bigger liability than reporting them [40]. Although pharmacists are in the best position to detect, correct and report medication errors, all HCPs, regardless of their role, need to be proactive in identifying system failures and to be responsible for reporting MEs as well as near-misses [40, 41]. A single medication error or a near-miss that did not even reach the patient may seem insignificant, but when the data on MEs is collected on a national level or even better, on a global level, it will become significant. Error reporting can influence practice guidelines, standards of procedures and manufacturing of products to prevent repeat errors and ultimately save unnecessary healthcare expenditures [40]. Unfortunately, while impressive developments in pharmacovigilance and MER have taken place in the West, not much has been achieved in the Southeast Asian countries [42]. The ADE reporting system in the ASEAN region still faces a number of challenges that need to be overcome in order to achieve a thorough understanding of ME. One such challenge is the serious lack of harmonization in ADE reporting systems across the countries of the region, resulting in differences in reporting standards. Moreover, due to a lack of awareness among HCPs, the general public and consumers, pharmacovigilance in this region is seen to be expensive and unnecessary, which is one factor in the serious lack of data on ME in this region.

### Limitations

There was no data regarding the incidence and types of ME in almost half of the Southeast Asian countries. Reconciliation error, preparation error and transcription error were inadequately evaluated. There was also only one study done on the Indonesian population, even though Indonesia is the most populous country in the region, comprising 40% of the total population residing in Southeast Asia. This means that the picture we currently have of the ME issue in Southeast Asia is very incomplete; furthermore countries with missing data are actually the ones that are less economically developed (which will probably translate to more ME). If data for these countries were available it would probably have made a significant difference to our current picture. Given the data currently available, therefore, it is not recommended to generalize the findings reported in this review for the whole of Southeast Asia. It must be noted that certain kinds of error such as “documentation” versus “administration” or “preparation” versus “dispensing” errors were not possible to be clearly distinguished. Furthermore, the interpretation and summarization of the collected data was hindered due to the differences in the approach taken by each author to report, define, interpret and classify data.

## Conclusions

Even though the studies focusing on ME in Southeast Asian countries are limited, and our results may not be generalized to the whole of the region, it remains clear that our initial concern about the incidence of ME in Southeast Asia has been validated. More studies need to be performed on this issue, therefore, especially in Brunei, Laos, Cambodia, Myanmar, Timor, Philippines and Indonesia. The root causes of ME may be deeper than one might expect, requiring fundamental changes in the health systems; one of these necessary changes is undoubtedly the need to include pharmacists in the health care team to utilize their expertise, since there is no one else better equipped to minimize medication errors than pharmacists.

In conclusion, this review has showed the insufficiency of medication error reporting and documentation in Southeast Asian countries and suggests that a collective and standardized effort is needed to improve the reporting and documentation of ME with the aim of minimising the occurrence of such errors.

## Supporting Information

S1 PRISMA Checklist PRISMA 2009 Checklist. (DOC)
