# Supplementary material for: Medication Errors in the Southeast Asian Countries: A Systematic Review
Source: PLoS One. 2015 Sep 4;10(9):e0136545. doi: 10.1371/journal.pone.0136545 (PMC4560405; doi:10.1371/journal.pone.0136545)
Supplement: S1 PRISMA Checklist — (DOC) [file pone.0136545.s001.doc]

| **Section/topic** | **#** | **Checklist item** | **Reported on page #** |
| --- | --- | --- | --- |
| **TITLE** | | |  |
| Title | 1 | Medication errors in the Southeast Asian countries: A systematic review | 1 |
| **ABSTRACT** | | |  |
| Structured summary | 2 | ***Background*:** Medication error (ME) is a worldwide issue, but most studies on ME have been undertaken in developed countries and very little is known about ME in Southeast Asian countries. This study aimed to systematically identify and review studies done on ME in Southeast Asian countries to identify common ME types, estimate its prevalence in this region and investigate the seriousness of this issue in Southeast Asia.  ***Methods*:** The literature relating to MEs in Southeast Asian countries was systematically reviewed in December 2014 by using; Embase®, Medline®, Pubmed®, ProQuest Central® and the CINAHL®. Inclusion criteria were studies (in any languages) that investigated the incidence and the contributing factors of ME in patients of all ages.  ***Results*:** The 17 included studies reported data on 6 of the 11 Southeast Asian countries; 5 studies in Singapore, 4 Malaysia, 3 Thailand, 3 Vietnam, 1 Philippines and 1 study in Indonesia. There was no data on MEs in Brunei, Laos, Cambodia, Myanmar and Timor. Of the 17 included studies, 11 measured administration errors, 4 focused on prescribing errors, 3 studies were done on preparation errors, 3 on dispensing errors and 2 on transcribing errors. There was only one study done on reconciliation error. Three studies were interventional. Reported error rates ranged from 15.2% to 88.6% for administration and 7% to 35.4% for prescribing error.  ***Discussion*:** The most frequently reported types of administration error were incorrect time, omission error and wrong dose. Staff shortage and hence heavy workload for nurses, doctor/ nurse distraction and misinterpretation of the prescription/medication chart were identified as contributing factors of ME. There is a serious lack of studies on this topic in this region which needs to be addressed if we are to fully understand and address the ME issue.  **Limitations**: There was no data regarding the incidence and types of ME in almost half of the Southeast Asian countries.  **Conclusion:** it is evident that our initial concern about the ME incidence in Southeast Asia has been validated, but these results may not be generalized to the whole of Southeast Asia. More studies need to be performed on this issue, therefore, especially in Brunei, Laos, Cambodia, Myanmar, Timor, Philippines and Indonesia. The root causes of ME may be deeper than one might expect, requiring fundamental changes in the health systems. | 2 |
| **INTRODUCTION** | | |  |
| Rationale | 3 | Medication error accounts for one third of preventable drug-related harm [7] and is the eighth leading cause of death in the US with more than 98,000 mortality annually [7], exceeded those from car accidents, breast cancer, or AIDS. It is a worldwide issue and Southeast Asian countries are no exception to this. The majority of the studies on this topic, however, have been carried out in developed nations such as North American and European countries, while the issue has been relatively neglected in Southeast Asia. | 3,4 |
| Objectives | 4 | In this systematic review of the literature, we focused on the types and prevalence of ME in Southeast Asian countries as certain aspects of the healthcare system in this region such as the ratio of healthcare professions to the general population and drug prescribing patterns are different from US and European Union countries | 4 |
| **METHODS** | | |  |
| Protocol and registration | 5 | N/a |  |
| Eligibility criteria | 6 | The following types of studies were included; randomized controlled trials, non-randomized controlled trials, longitudinal studies, cohort or case–control studies, and descriptive studies. There was no limitation imposed on the year of publication of the studies. Reviews, letters, case studies, conference papers, opinions, reports or editorial papers were not included, however.  The search strategy included human studies of all languages, on patients of all ages. | 4,5 |
| Information sources | 7 | The literature relating to MEs in Southeast Asian countries was systematically reviewed in December 2014 by using; Embase®, Medline®, Pubmed®, ProQuest Central® and the CINAHL®. | 5 |
| Search | 8 | PubMed: ((((((((Medication error) AND Timor)) OR ((medication error) AND Indonesia)) OR ((medication error) AND Myanmar)) OR ((medication error) AND Cambodia)) OR ((medication error) AND Philippines)) OR ((medication error) AND Laos)) OR ((medication error) AND Vietnam) | 5 |
| Study selection | 9 | Identification, title and abstract screening, full text eligibility assessment, included in systematic review. | 4 |
| Data collection process | 10 | The literature relating to MEs in Southeast Asian countries was collected using; Embase®, Medline®, Pubmed®, ProQuest Central® and the CINAHL®. |  |
| Data items | 11 | The literature relating to MEs in Southeast Asian countries till Dec 2014 was included in this study |  |
| Risk of bias in individual studies | 12 | It was not performed in this study because the we had also included longitudinal studies and descriptive studies. Furthermore, we do not perform meta-analysis for this study |  |
| Summary measures | 13 | Not applicable because we do not perform meta-analysis for this study |  |
| Synthesis of results | 14 | Not applicable because we do not perform meta-analysis for this study |  |

Page 1 of 2

| **Section/topic** | **#** | **Checklist item** | **Reported on page #** |
| --- | --- | --- | --- |
| Risk of bias across studies | 15 | Not applicable because we do not perform meta-analysis for this study |  |
| Additional analyses | 16 | Not applicable because we do not perform meta-analysis for this study |  |
| **RESULTS** | | |  |
| Study selection | 17 | Total number of Articles initially identified: 1274  32 Duplicates excluded.  1242 Titles & Abstracts screened  1217 Titles excluded due to:  508: Not relevant to the specific country.  709: Not relevant to the topic.  25 Full text articles assessed for eligibility  8 Full text articles excluded due to:  3: Irrelevant focus.  5: case study, report, opinion, editorial paper, etc…  17 Full text articles included | 4 |
| Study characteristics | 18 | | **Studies done on administration error** | | | | | | | | | --- | --- | --- | --- | --- | --- | --- | --- | | **#** | **Setting** | **Methodology** | | **Study duration** | **Sample** | **Results** | **Reference** | | 1 | Malaysia  (Paediatric ward of a teaching hospital) | Direct observational study | | 10 days over 10 weeks | 857 administrations | Incorrect dose form: 2%  Incorrect time: 30%  Incorrect technique: 9%  Unauthorized drug: 7%  Omission error: 17% due to out of stock  Incorrect dose: 12%  Incorrect preparation: 27% | [12] | | 2 | Malaysia  (tertiary care hospital) | Prospective observational study | | 3 months | 349 IV drugs which were prepared and administered by the staff nurses to the patients. | Wrong time: 42.1% Wrong technique: 19.5% Wrong admin rate: 85.1% | [13] | | 3 | Malaysia  (haematology ward of a teaching hospital in Malaysia) | Prospective study that involved direct, undisguised observations of drug  Administrations | | 15 days | 1118 total opportunities for error | Incorrect drug: 0.7 % Extra dose: 2.2% Administration of expired medications: 2.2% Incorrect rate: 5.9% Omission: 10.4%  Incorrect dose: 10.4% Incorrect drug preparation: 10.4% Unauthorized drug: 14.1% Incorrect technique: 16.3% Incorrect time: 25.2% | [14] | | 4 | Singapore (public sector and private practice anaesthesiologists in Singapore) | Survey | | 1 month | 350 survey forms | Misidentification of the ampoule: 53% Misidentification of syringes: 45% | [15] | | 5 | Singapore  (two acute care hospitals) | Descriptive, prospective design | | 12 weeks | 21043 opportunity for error (doses given or doses orders but omitted)  An opportunity for error  included any dose given plus doses ordered to be given but  omitted | Of the 140 participants, only 10% (14/140) were not  observed to encounter any distractions during medication  administration, while 90% (126/140) were distracted during  the observations | [16] | | 6 | Vietnam  (2 public hospitals) | Direct observational study | | 7 days | 229 insulin doses (204 subcutaneous and 25 infusions) | Delayed dose: 10.4% Early dose: 7.4% Administration technique error: 3.1% Omission: 2.6% | [17] | | 7 | Vietnam  (6 wards in 2 urban public hospitals) | Prospective observational | | 3 months | 5271 doses administered | Wrong administration technique: 23.5%  Wrong preparation technique: 15.7%  Omission: 2.3%  Wrong dose: 1.8% | [18] | | 8 | Indonesia-Bali  (Geriatric ward in a public teaching hospital) | Prospective study | | 20 weeks | 7662 doses | Administration errors: 59% | [19] | | 9 | Philippines  (University-based tertiary hospital) | Questionnaire | |  | 329 questionnaires | Missed dose: 41.94% Wrong time: 40.32% | [20] | | 10 | Thailand  (7 university hospitals,  5 tertiary care hospitals,  4 secondary care hospitals, 4 primary care hospitals) | Prospective data collection | | 18 months | 202699 anaesthesia cases | Wrong drug: 48.8% Incorrect dose: 29.3% | [21] | | 11 | Thailand (Queen Sirikit National Institute of Child Health) | Retrospective study (screening medication errors documents and reports) | | 15 months | Medication errors in ward documented in standard reporting forms based on the 32106 admissions | Admin error: 15.22 % Wrong time: 2.17% Omission 1.24 % Wrong strength: 1.86% Unauthorized drug: 0% Wrong patient : 2.48% Extra dose: 3.73% Wrong dose form: 3.73% | [22] | | **Studies done on dispensing error** | | | | | | | | | 1 | Indonesia-Bali (Geriatric ward in a public teaching hospital) | | Prospective study | 20 weeks | 7662 doses | Dispensing errors: 14% Omission: 39.6% | [19] | | 2 | Thailand (Queen Sirikit National Institute of child health) | | Retrospective study (screening ME documents and reports) | 15 months | 32105 | Dispensing: 34.78% | [22] | | **Studies done on prescribing error** | | | | | | | | | 1 | Malaysia  (outpatient pharmacy in a teaching hospital in Kelantan) | | Retrospective study. (screening prescriptions) | 1 month | 1601 prescriptions for geriatrics | Pharmaceutical (stability, ingredient, technique): 0.99%  Clinical errors (dose,frequency, interaction,allergy): 8.68% | [23] | | 2 | Indonesia  (Geriatric ward in a public teaching hospital in Bali) | | Prospective study | 20 weeks | 7662 doses | Prescribing errors: 7% | [19] | | 3 | Singapore  (Paediatric unit in a university teaching hospital) | | Prospective cohort study | 4 months | 4274 paediatric prescriptions | Under-dose: 64% No frequency specified: 21%  Overdose: 8% | [24] | | 4 | Thailand  (Queen Sirikit National Institute of child health) | | Retrospective study (screening ME docs and reports) | 15 months | 32105 | Prescription error: 35.4% Wrong dose: 25.78% Wrong choice: 3.73 % Known allergy: 0.62% | [22] | | **Studies done on transcribing error** | | | | | | | | | 1 | Malaysia  (outpatient pharmacy in a teaching hospital (HUSM) in Kelantan) | | Retrospective study. (screening prescriptions) | 1 month | 1601 prescriptions for geriatrics | Miswriting patient particulars: 70.22% | [23] | | 2 | Indonesia  (Geriatric ward in a public teaching hospital in Bali) | | Prospective study | 20 weeks | 7662 doses | Transcription errors: 15% | [19] | | **Studies done on preparation error** | | | | | | | | | 1 | Malaysia  (tertiary care hospital) | | Prospective observational study | 3 months | 349 IV drugs prepared and administered by nurses | Preparation errors :32.8% Wrong amount of diluents: 54.5% | [13] | | 2 | Vietnam  (Two large public hospitals in Vietnam) | | Direct observational study | 7 days | 229 insulin doses (204 subcutaneous and 25 infusions) | Incorrect preparation technique: 22.7% | [17] | | 3 | Vietnam  (two urban public hospitals in Vietnam) | | Prospective observational | 3 months | 5271 oral and IV doses administered | Wrong preparation technique: 15.7% | [18] | | **Studies done on reconciliation error** | | | | | | | | | 1 | Singapore  (Tan Tock Seng Hospital) | | Descriptive | NA | Reconciliation forms created by pharmacy staff for each patient admitted | Transcription error: 36.5% Prescribers missing out medications from their list: 61.65% Wrong or incomplete regimen: 25.4% | [25] | | 7,8,9 |
| Risk of bias within studies | 19 | Not applicable |  |
| Results of individual studies | 20 | Not applicable |  |
| Synthesis of results | 21 | Not applicable |  |
| Risk of bias across studies | 22 | Not applicable |  |
| Additional analysis | 23 | Not applicable |  |
| **DISCUSSION** | | |  |
| Summary of evidence | 24 | **Administration error**  Medication administration error is defined as any discrepancy between the medicine given to the patient and the prescriber's medication order as written on the patient's chart or manufacturers' preparation/administration instructions [26]. Eleven of the seventeen studies discussed administration errors. The reported administration error rates ranged from 15.2%, to 88.6% [13, 19, 22]. “Wrong time” (early or delayed doses) was the most frequently cited type of administration error [12-14, 17, 20, 22], along with omission error, where the dose was not administered at all [12, 17, 20, 22, 23]. The next most frequently mentioned type of administration error was “wrong dose” [12, 14, 21, 22].  Ong et al. [13] who focused on IV drug administration errors reported that the most frequent administration errors were mistakes in the technique of IV administration, along with the medication being administered at the wrong rate (usually too fast).  Chua et al. [12] who investigated administration error in paediatric wards, reported that administration errors are more prevalent in oncology wards since the medicines are more complex. The same study also mentioned that liquid dose forms are more prone to administration error as compared to solid dose forms because of the measurement errors that occur during the measurement of the volume required for liquid doses.  The other frequently reported reasons behind administration error were: heavy workload of nurses (due to nurse shortage) [12-14, 19, 20, 27], lack of knowledge [12, 14], stock shortage [12, 23] and calculation error [12, 14].  **Dispensing error**  Dispensing errors happen when the medication dispensed/delivered by the pharmacy is not compatible with the order written in the prescription by the doctor [28]. Of the seventeen studies, four investigated dispensing error; with one retrospective, one prospective and two observational studies. Ernawati et al. reported an error incidence of 14%, Nguyen HT et al. reported a rate of 22.7%, while Ong et al. and Sangtawesin et al. reported roughly equal error incidence (32.8% and 34.78% respectively). Ernawati et al. reported omission as the main type of dispensing error followed by, in order of decreasing frequency, labelling errors, wrong quantity of drug, wrong dose, duplication, wrong drug, drug dispensed although not ordered, wrong dosage form and wrong patient. Additional types of dispensing error mentioned by Ong et al. were: wrong diluents or wrong amount of diluents, exceeding stability time after reconstitution, improper mixing.  **Prescribing error**  Prescribing error is defined as any error in the process of prescribing the medication that leads to (or has the potential to lead to) patient harm [29]. The error rates reported varied greatly; the highest rate of prescribing error was reported by Sangtawesin et al., which was 35.4%, while the lowest error rate was 7% reported by Ernawati et al. The wrong dose was the most frequently mentioned type of prescribing error [19, 22-24]. Goh et al., however, stated that under-dosing was significantly more prevalent that overdosing and also that paediatricians made fewer prescribing mistakes than non-paediatricians (18.4% vs 25.1%) [24].  **Preparation error**  Errors that occur during the preparation of a medicine could either happen in the pharmacy - for example when the pharmacist prepares an incorrect dilution for an oral syrup, or by the nurse in the ward when reconstituting an intravenous solution or crushing modified release tablets for oral tube administration. Some of the studies, however, did not really specify whether these errors occurred in the pharmacy or in the ward; in this review, therefore, any medication error that occurred in the process of preparation were classified as “preparation error” regardless of the health-professional responsible for it. Three of the seventeen included studies focused on preparation errors by either the pharmacist or the nurse. Two of these were conducted in Vietnam [17, 18] and one in Malaysia [13]. The common types of errors that happened in the preparation stage were use of the wrong technique or the wrong diluents.  **Transcribing error**  According to Fahimi et al. “Transcription error is a specific type of medication error and is due to data entry error that is commonly made by the human operators” [30]. Two of the included studies investigated transcribing error [19, 23]; one in Malaysia and the other in Indonesia, with very different error rates reported: 15% [19] versus 70.22% [23]. Ernawati et al. reported that 35.2% of these errors involved drugs needed by patients not being transcribed either onto the medication chart or drug order form, or into the nurse’s log book; resulting in seven drug omissions in the administration stage and two delayed administrations.  **Reconciliation error**  The Australian Commission on Safety and Quality in Healthcare defines medication reconciliation as “a formal process of obtaining and verifying a complete and accurate list of each patient’s current medicines”. Unfortunately there was only one study focused on errors that occur in this stage of patient care. This was performed in Singapore and reported a transcription error rate of 36.5%, which was mainly due to prescribers missing out medications from their list (61.6%) and an incorrect or incomplete regimen being transcribed (25.4%) [25].  **Interventional studies**  Three studies investigated interventions used to reduce MEs (Table 3). Sanguansak et al. examined the use of formulary script instead of handwritten prescriptions and reported a significant decrease in prescribing errors such as drug name error, incorrect strength and incorrect route [31]. Nguyen et al. investigated the effect of a clinical pharmacist led training programme on intravenous medication errors, with a significant reduction of ME (from 64.0% to 48.9%) being observed in the intensive care unit (ICU) in cases where education had been provided by the pharmacist. Not all the interventional studies showed positive outcomes, however. In the study performed by Choo et al., an inpatient electronic medication record system was the intervention used to reduce ME, but the system was found to have little effect [32].  **Types of errors**  Medication errors are typically classified into a few broad groups according to the stage at which the ME occurred (such as dispensing error, administration error, etc.). Although this approach can be useful, it does not help much when it comes to the prevention of MEs since it is important to know the exact types of errors that occur in practice. In the Southeast Asian countries, the wrong dose was the most common type of error [12, 14, 17, 19, 21-24, 31], with the reported rate of dose error – ranging between 12% [12] and 72% [24]. Other types of errors reported were:  Omission error [14, 17, 19, 20, 22, 25]  Incorrect time [12-14, 20, 22]  Wrong drug [14, 15, 17, 21, 22]  Incorrect administration technique [12-14, 17]  Wrong dose form [12, 22, 25]  Medications involved  Most studies did not mention the medications involved in the ME, although some did mention the name of the drug, while some others mentioned the drug class. The most frequently reported class of drugs related to ME was antibiotics [12, 13, 21, 22]. Other common medicines involved were opioids [15, 21], corticosteroids [12, 22] and muscle relaxants [15, 21].  **Severity of the medication errors**  Unfortunately, in most of the studies, the clinical consequences of the reported MEs were not investigated. Nguyen et al. reported that 23.5% of the doses were judged to have potentially moderate outcomes and 3.5% potentially severe outcomes. Sangtawesin et al., who classified MEs in term of their severity, reported two clinically significant MEs, an over-dosage and a wrong dose for administration [22]. Thanoo et al., meanwhile, reported that 34.1% (14 out of 41 incidents) of MEs led to short term, mild to severe physiological effects, with all of the affected patients making a complete recovery, apart for one who died [21].  Generally speaking, however, the identified MEs led to interventions by researchers due to ethical reasons and hence did not lead to any clinically significant consequences for the patient.  **Factors contributing to medication errors**  The factors contributing to MEs were reported in 15 studies [12-16, 19-25, 27, 31, 32]. The most common ones are listed below; it is important to remember, however, that MEs usually arise from poorly designed work environments and systems rather than the individual performance of a single practitioner [33].  Staff shortage/high workload [12-14, 19-21, 27]  Nurse/doctor distraction [12, 31, 32]  Incorrect interpretation of prescription/medication chart [12, 14, 31]  Lack of knowledge [12, 14]  Lack of experience [20, 24]  It must be noted, however, that, while not the focus of this review, patients themselves may also contribute to the incidence of medication errors due to a number of reasons such as forgetfulness, lack of cooperation or confusion. | 9,10,11,12  ,13 |
| Limitations | 25 | There was no data regarding the incidence and types of ME in almost half of the Southeast Asian countries. Reconciliation error, preparation error and transcription error were inadequately evaluated; The picture we currently have of the ME issue in Southeast Asia is hence very incomplete; countries with missing data are actually the ones that are less economically developed (which will probably translate to more ME), this leads to a huge bias when interpreting the data because if data for these countries were available it would have made a significant difference in our current picture. Therefore, it is not recommended to generalize the findings reported in this review for the whole Southeast Asia. Furthermore, the interpretation and summarization of the collected data was hindered due to the differences in the approach taken by each author to report, define, interpret and classify data. | 17 |
| Conclusions | 26 | The number of studies done on ME in this region was unfortunately very limited (17 only); of the 11 countries that make up Southeast Asia, only six had reported data on MEs, which could be due to the lack of MERS in this region. From the included studies, however, it is evident that our initial concern about the ME incidence in Southeast Asia has been validated, but these results may not be generalized to the whole of Southeast Asia. More studies need to be performed on this issue, therefore, especially in Brunei, Laos, Cambodia, Myanmar, Timor, Philippines and Indonesia. The root causes of ME may be deeper than one might expect, requiring fundamental changes in the health systems; one of these necessary changes is undoubtedly the need to include pharmacists in the health care team to utilize their expertise, since there is no one else better equipped to minimize medication errors than pharmacists.  In conclusion, this review showed the insufficiency of medication error reporting and documentation in Southeast Asian countries and suggests that a collective and standardized effort is needed to improve the reporting and documentation of ME with the aim to prevent its recurrence | 18 |
| **FUNDING** | | |  |
| Funding | 27 | This systematic review received no funding |  |

*From:*  Moher D, Liberati A, Tetzlaff J, Altman DG, The PRISMA Group (2009). Preferred Reporting Items for Systematic Reviews and Meta-Analyses: The PRISMA Statement. PLoS Med 6(6): e1000097. doi:10.1371/journal.pmed1000097

For more information, visit: **www.prisma-statement.org**.

Page 2 of 2
